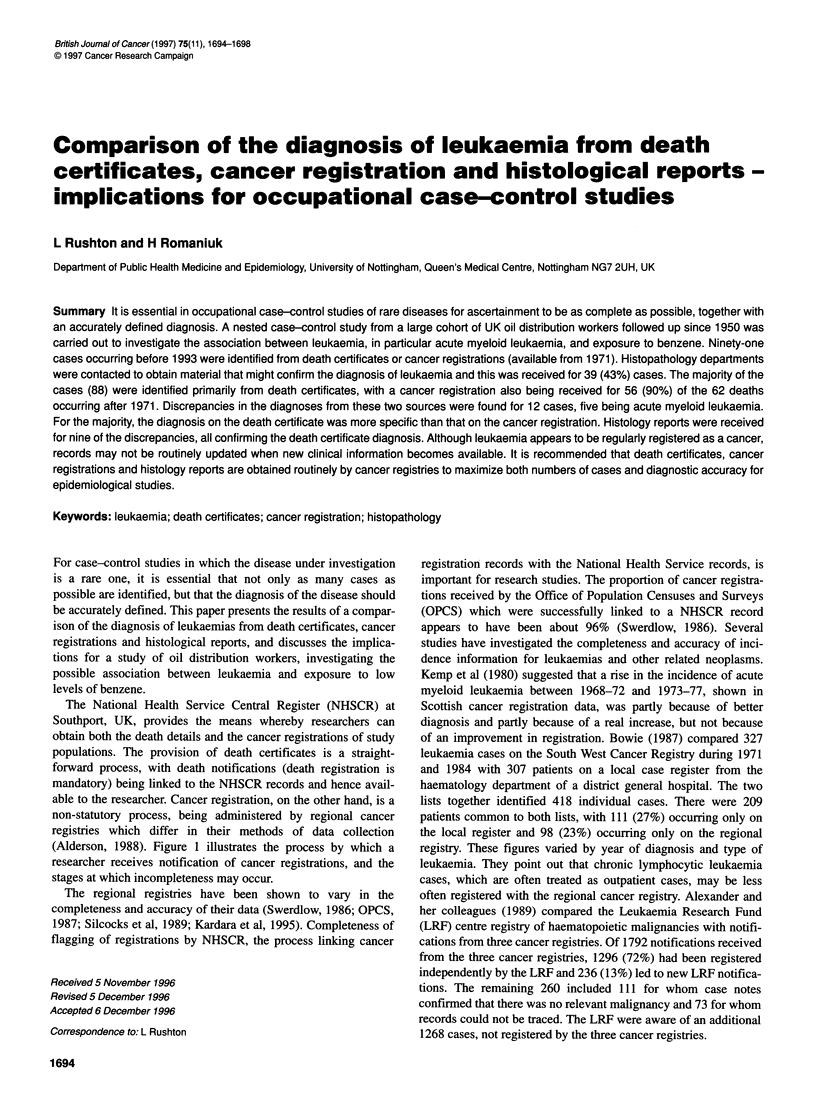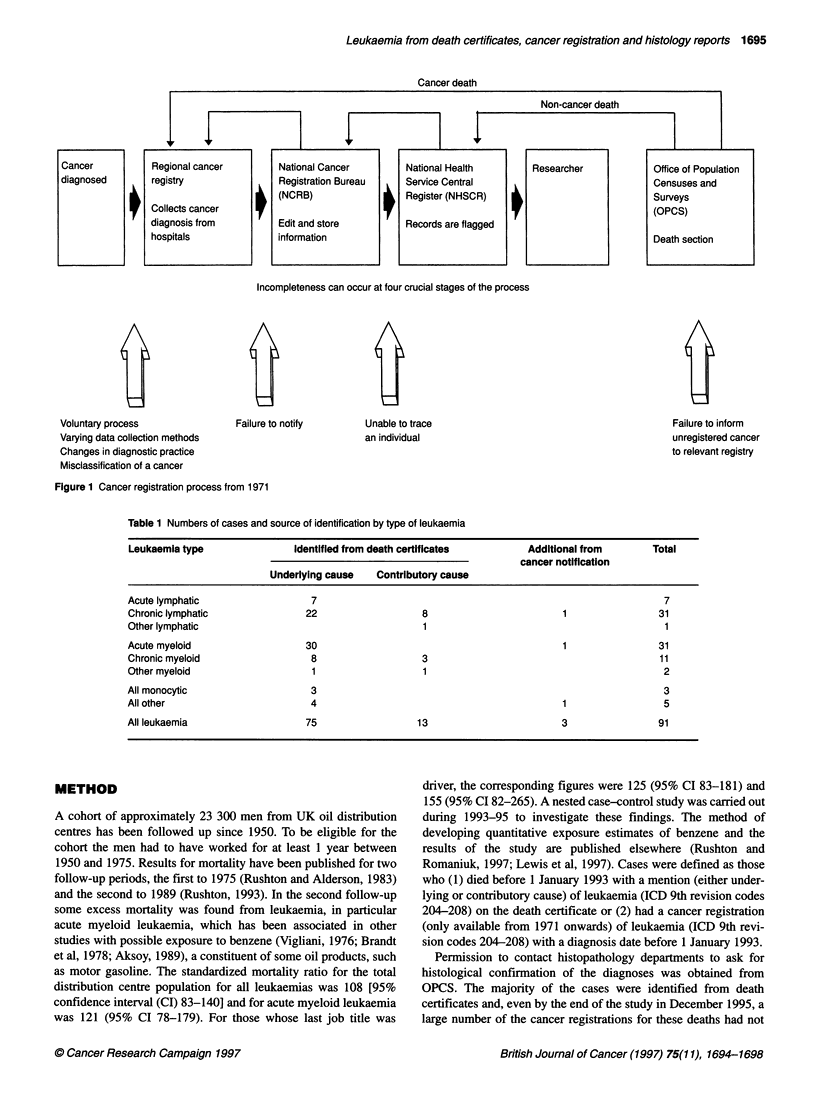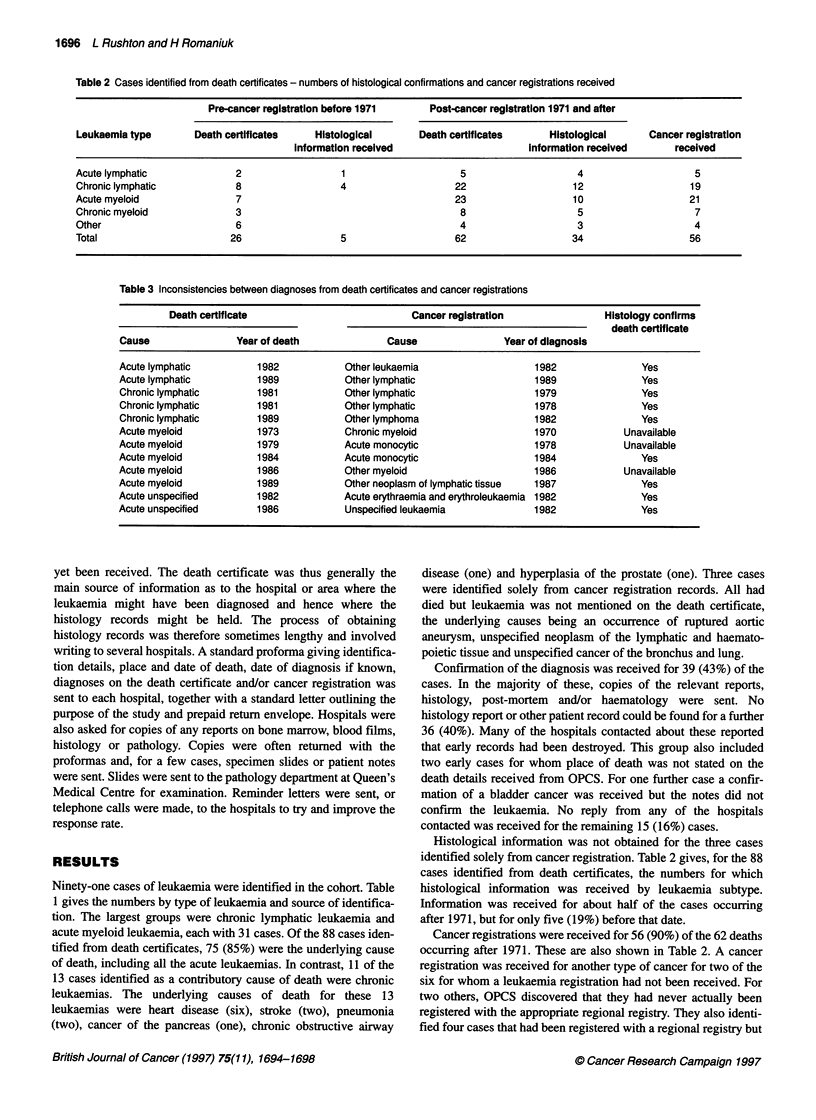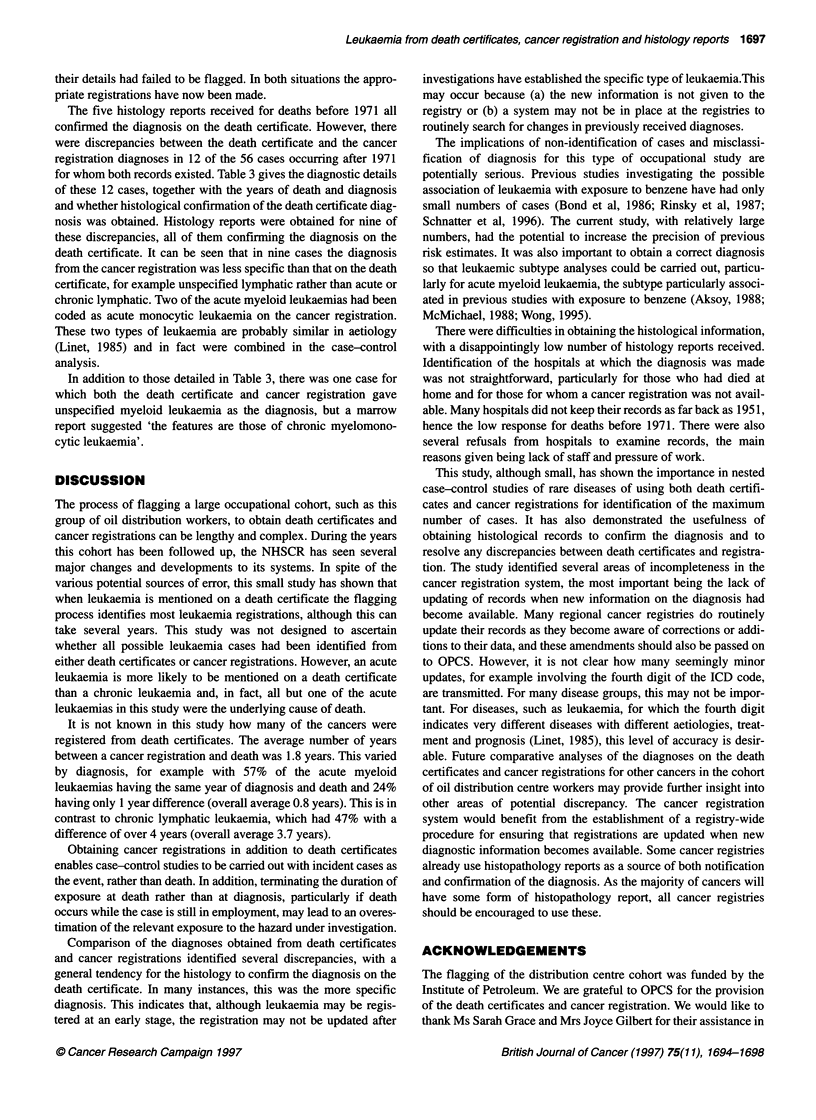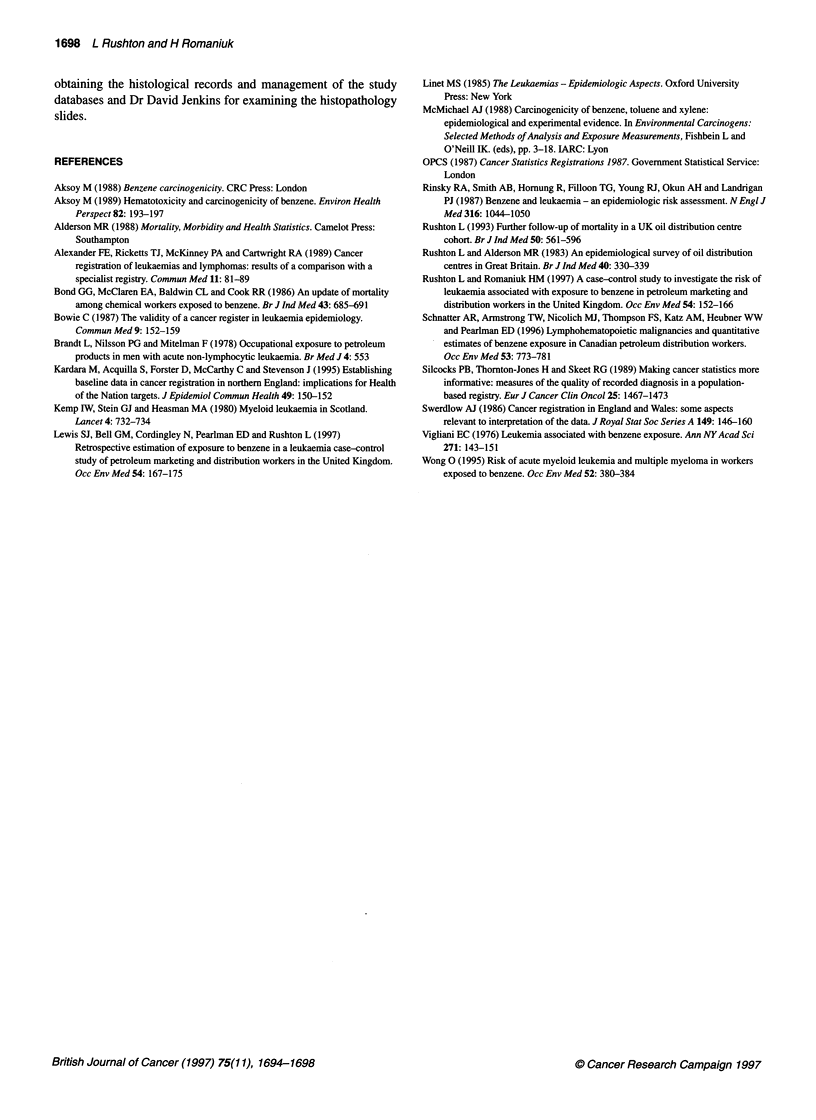# Comparison of the diagnosis of leukaemia from death certificates, cancer registration and histological reports - implications for occupational case-control studies.

**DOI:** 10.1038/bjc.1997.288

**Published:** 1997

**Authors:** L. Rushton, H. Romaniuk

**Affiliations:** Department of Public Health and Epidemiology, University of Nottingham, Queen's Medical Centre, UK.

## Abstract

It is essential in occupational case-control studies of rare diseases for ascertainment to be as complete as possible, together with an accurately defined diagnosis. A nested case-control study from a large cohort of UK oil distribution workers followed up since 1950 was carried out to investigate the association between leukaemia, in particular acute myeloid leukaemia, and exposure to benzene. Ninety-one cases occurring before 1993 were identified from death certificates or cancer registrations (available from 1971). Histopathology departments were contacted to obtain material that might confirm the diagnosis of leukaemia and this was received for 39 (43%) cases. The majority of the cases (88) were identified primarily from death certificates, with a cancer registration also being received for 56 (90%) of the 62 deaths occurring after 1971. Discrepancies in the diagnoses from these two sources were found for 12 cases, five being acute myeloid leukaemia. For the majority, the diagnosis on the death certificate was more specific than that on the cancer registration. Histology reports were received for nine of the discrepancies, all confirming the death certificate diagnosis. Although leukaemia appears to be regularly registered as a cancer, records may not be routinely updated when new clinical information becomes available. It is recommended that death certificates, cancer registrations and histology reports are obtained routinely by cancer registries to maximize both numbers of cases and diagnostic accuracy for epidemiological studies.


British Journal of Cancer (1997) 75(11), 1694–1698
© 1997 Cancer Research Campaign

### Comparison of the diagnosis of leukaemia from death certificates, cancer registration and histological reports – implications for occupational case–control studies

**L Rushton and H Romaniuk**

Department of Public Health Medicine and Epidemiology, University of Nottingham, Queen's Medical Centre, Nottingham NG7 2UH, UK

**Summary** It is essential in occupational case–control studies of rare diseases for ascertainment to be as complete as possible, together with an accurately defined diagnosis. A nested case–control study from a large cohort of UK oil distribution workers followed up since 1950 was carried out to investigate the association between leukaemia, in particular acute myeloid leukaemia, and exposure to benzene. Ninety-one cases occurring before 1993 were identified from death certificates or cancer registrations (available from 1971). Histopathology departments were contacted to obtain material that might confirm the diagnosis of leukaemia and this was received for 39 (43%) cases. The majority of the cases (88) were identified primarily from death certificates, with a cancer registration also being received for 56 (90%) of the 62 deaths occurring after 1971. Discrepancies in the diagnoses from these two sources were found for 12 cases, five being acute myeloid leukaemia. For the majority, the diagnosis on the death certificate was more specific than that on the cancer registration. Histology reports were received for nine of the discrepancies, all confirming the death certificate diagnosis. Although leukaemia appears to be regularly registered as a cancer, records may not be routinely updated when new clinical information becomes available. It is recommended that death certificates, cancer registrations and histology reports are obtained routinely by cancer registries to maximize both numbers of cases and diagnostic accuracy for epidemiological studies.

**Keywords:** leukaemia; death certificates; cancer registration; histopathology

For case–control studies in which the disease under investigation is a rare one, it is essential that not only as many cases as possible are identified, but that the diagnosis of the disease should be accurately defined. This paper presents the results of a comparison of the diagnosis of leukaemias from death certificates, cancer registrations and histological reports, and discusses the implications for a study of oil distribution workers, investigating the possible association between leukaemia and exposure to low levels of benzene.

The National Health Service Central Register (NHSCR) at Southport, UK, provides the means whereby researchers can obtain both the death details and the cancer registrations of study populations. The provision of death certificates is a straightforward process, with death notifications (death registration is mandatory) being linked to the NHSCR records and hence available to the researcher. Cancer registration, on the other hand, is a non-statutory process, being administered by regional cancer registries which differ in their methods of data collection (Alderson, 1988). Figure 1 illustrates the process by which a researcher receives notification of cancer registrations, and the stages at which incompleteness may occur.

The regional registries have been shown to vary in the completeness and accuracy of their data (Swerdlow, 1986; OPCS, 1987; Silcocks et al, 1989; Kardara et al, 1995). Completeness of flagging of registrations by NHSCR, the process linking cancer registration records with the National Health Service records, is important for research studies. The proportion of cancer registrations received by the Office of Population Censuses and Surveys (OPCS) which were successfully linked to a NHSCR record appears to have been about 96% (Swerdlow, 1986). Several studies have investigated the completeness and accuracy of incidence information for leukaemias and other related neoplasms. Kemp et al (1980) suggested that a rise in the incidence of acute myeloid leukaemia between 1968–72 and 1973–77, shown in Scottish cancer registration data, was partly because of better diagnosis and partly because of a real increase, but not because of an improvement in registration. Bowie (1987) compared 327 leukaemia cases on the South West Cancer Registry during 1971 and 1984 with 307 patients on a local case register from the haematology department of a district general hospital. The two lists together identified 418 individual cases. There were 209 patients common to both lists, with 111 (27%) occurring only on the local register and 98 (23%) occurring only on the regional registry. These figures varied by year of diagnosis and type of leukaemia. They point out that chronic lymphocytic leukaemia cases, which are often treated as outpatient cases, may be less often registered with the regional cancer registry. Alexander and her colleagues (1989) compared the Leukaemia Research Fund (LRF) centre registry of haematopoietic malignancies with notifications from three cancer registries. Of 1792 notifications received from the three cancer registries, 1296 (72%) had been registered independently by the LRF and 236 (13%) led to new LRF notifications. The remaining 260 included 111 for whom case notes confirmed that there was no relevant malignancy and 73 for whom records could not be traced. The LRF were aware of an additional 1268 cases, not registered by the three cancer registries.

*Received 5 November 1996*
*Revised 5 December 1996*
*Accepted 6 December 1996*

*Correspondence to:* L Rushton

**Figure 1** Cancer registration process from 1971

**Table 1** Numbers of cases and source of identification by type of leukaemia

| Leukaemia type | Identified from death certificates | | Additional from cancer notification | Total |
|---|---|---|---|---|
| | Underlying cause | Contributory cause | | |
| Acute lymphatic | 7 | | | 7 |
| Chronic lymphatic | 22 | 8 | 1 | 31 |
| Other lymphatic | | 1 | | 1 |
| Acute myeloid | 30 | | 1 | 31 |
| Chronic myeloid | 8 | 3 | | 11 |
| Other myeloid | 1 | 1 | | 2 |
| All monocytic | 3 | | | 3 |
| All other | 4 | | 1 | 5 |
| All leukaemia | 75 | 13 | 3 | 91 |

### METHOD

A cohort of approximately 23 300 men from UK oil distribution centres has been followed up since 1950. To be eligible for the cohort the men had to have worked for at least 1 year between 1950 and 1975. Results for mortality have been published for two follow-up periods, the first to 1975 (Rushton and Alderson, 1983) and the second to 1989 (Rushton, 1993). In the second follow-up some excess mortality was found from leukaemia, in particular acute myeloid leukaemia, which has been associated in other studies with possible exposure to benzene (Vigliani, 1976; Brandt et al, 1978; Aksoy, 1989), a constituent of some oil products, such as motor gasoline. The standardized mortality ratio for the total distribution centre population for all leukaemias was 108 [95% confidence interval (CI) 83–140] and for acute myeloid leukaemia was 121 (95% CI 78–179). For those whose last job title was driver, the corresponding figures were 125 (95% CI 83–181) and 155 (95% CI 82–265). A nested case–control study was carried out during 1993–95 to investigate these findings. The method of developing quantitative exposure estimates of benzene and the results of the study are published elsewhere (Rushton and Romaniuk, 1997; Lewis et al, 1997). Cases were defined as those who (1) died before 1 January 1993 with a mention (either underlying or contributory cause) of leukaemia (ICD 9th revision codes 204–208) on the death certificate or (2) had a cancer registration (only available from 1971 onwards) of leukaemia (ICD 9th revision codes 204–208) with a diagnosis date before 1 January 1993.

Permission to contact histopathology departments to ask for histological confirmation of the diagnoses was obtained from OPCS. The majority of the cases were identified from death certificates and, even by the end of the study in December 1995, a large number of the cancer registrations for these deaths had not

© *Cancer Research Campaign 1997*

Table 2 Cases identified from death certificates – numbers of histological confirmations and cancer registrations received

| Leukaemia type | Pre-cancer registration before 1971: Death certificates | Pre-cancer registration before 1971: Histological information received | Post-cancer registration 1971 and after: Death certificates | Post-cancer registration 1971 and after: Histological information received | Cancer registration received |
|---|---|---|---|---|---|
| Acute lymphatic | 2 | 1 | 5 | 4 | 5 |
| Chronic lymphatic | 8 | 4 | 22 | 12 | 19 |
| Acute myeloid | 7 | | 23 | 10 | 21 |
| Chronic myeloid | 3 | | 8 | 5 | 7 |
| Other | 6 | | 4 | 3 | 4 |
| Total | 26 | 5 | 62 | 34 | 56 |

Table 3 Inconsistencies between diagnoses from death certificates and cancer registrations

| Death certificate | | Cancer registration | | Histology confirms death certificate |
|---|---|---|---|---|
| Cause | Year of death | Cause | Year of diagnosis | |
| Acute lymphatic | 1982 | Other leukaemia | 1982 | Yes |
| Acute lymphatic | 1989 | Other lymphatic | 1989 | Yes |
| Chronic lymphatic | 1981 | Other lymphatic | 1979 | Yes |
| Chronic lymphatic | 1981 | Other lymphatic | 1978 | Yes |
| Chronic lymphatic | 1989 | Other lymphoma | 1982 | Yes |
| Acute myeloid | 1973 | Chronic myeloid | 1970 | Unavailable |
| Acute myeloid | 1979 | Acute monocytic | 1978 | Unavailable |
| Acute myeloid | 1984 | Acute monocytic | 1984 | Yes |
| Acute myeloid | 1986 | Other myeloid | 1986 | Unavailable |
| Acute myeloid | 1989 | Other neoplasm of lymphatic tissue | 1987 | Yes |
| Acute unspecified | 1982 | Acute erythraemia and erythroleukaemia | 1982 | Yes |
| Acute unspecified | 1986 | Unspecified leukaemia | 1982 | Yes |

yet been received. The death certificate was thus generally the main source of information as to the hospital or area where the leukaemia might have been diagnosed and hence where the histology records might be held. The process of obtaining histology records was therefore sometimes lengthy and involved writing to several hospitals. A standard proforma giving identification details, place and date of death, date of diagnosis if known, diagnoses on the death certificate and/or cancer registration was sent to each hospital, together with a standard letter outlining the purpose of the study and prepaid return envelope. Hospitals were also asked for copies of any reports on bone marrow, blood films, histology or pathology. Copies were often returned with the proformas and, for a few cases, specimen slides or patient notes were sent. Slides were sent to the pathology department at Queen's Medical Centre for examination. Reminder letters were sent, or telephone calls were made, to the hospitals to try and improve the response rate.

### RESULTS

Ninety-one cases of leukaemia were identified in the cohort. Table 1 gives the numbers by type of leukaemia and source of identification. The largest groups were chronic lymphatic leukaemia and acute myeloid leukaemia, each with 31 cases. Of the 88 cases identified from death certificates, 75 (85%) were the underlying cause of death, including all the acute leukaemias. In contrast, 11 of the 13 cases identified as a contributory cause of death were chronic leukaemias. The underlying causes of death for these 13 leukaemias were heart disease (six), stroke (two), pneumonia (two), cancer of the pancreas (one), chronic obstructive airway disease (one) and hyperplasia of the prostate (one). Three cases were identified solely from cancer registration records. All had died but leukaemia was not mentioned on the death certificate, the underlying causes being an occurrence of ruptured aortic aneurysm, unspecified neoplasm of the lymphatic and haematopoietic tissue and unspecified cancer of the bronchus and lung.

Confirmation of the diagnosis was received for 39 (43%) of the cases. In the majority of these, copies of the relevant reports, histology, post-mortem and/or haematology were sent. No histology report or other patient record could be found for a further 36 (40%). Many of the hospitals contacted about these reported that early records had been destroyed. This group also included two early cases for whom place of death was not stated on the death details received from OPCS. For one further case a confirmation of a bladder cancer was received but the notes did not confirm the leukaemia. No reply from any of the hospitals contacted was received for the remaining 15 (16%) cases.

Histological information was not obtained for the three cases identified solely from cancer registration. Table 2 gives, for the 88 cases identified from death certificates, the numbers for which histological information was received by leukaemia subtype. Information was received for about half of the cases occurring after 1971, but for only five (19%) before that date.

Cancer registrations were received for 56 (90%) of the 62 deaths occurring after 1971. These are also shown in Table 2. A cancer registration was received for another type of cancer for two of the six for whom a leukaemia registration had not been received. For two others, OPCS discovered that they had never actually been registered with the appropriate regional registry. They also identified four cases that had been registered with a regional registry but

© Cancer Research Campaign 1997

their details had failed to be flagged. In both situations the appropriate registrations have now been made.

The five histology reports received for deaths before 1971 all confirmed the diagnosis on the death certificate. However, there were discrepancies between the death certificate and the cancer registration diagnoses in 12 of the 56 cases occurring after 1971 for whom both records existed. Table 3 gives the diagnostic details of these 12 cases, together with the years of death and diagnosis and whether histological confirmation of the death certificate diagnosis was obtained. Histology reports were obtained for nine of these discrepancies, all of them confirming the diagnosis on the death certificate. It can be seen that in nine cases the diagnosis from the cancer registration was less specific than that on the death certificate, for example unspecified lymphatic rather than acute or chronic lymphatic. Two of the acute myeloid leukaemias had been coded as acute monocytic leukaemia on the cancer registration. These two types of leukaemia are probably similar in aetiology (Linet, 1985) and in fact were combined in the case–control analysis.

In addition to those detailed in Table 3, there was one case for which both the death certificate and cancer registration gave unspecified myeloid leukaemia as the diagnosis, but a marrow report suggested 'the features are those of chronic myelomonocytic leukaemia'.

### DISCUSSION

The process of flagging a large occupational cohort, such as this group of oil distribution workers, to obtain death certificates and cancer registrations can be lengthy and complex. During the years this cohort has been followed up, the NHSCR has seen several major changes and developments to its systems. In spite of the various potential sources of error, this small study has shown that when leukaemia is mentioned on a death certificate the flagging process identifies most leukaemia registrations, although this can take several years. This study was not designed to ascertain whether all possible leukaemia cases had been identified from either death certificates or cancer registrations. However, an acute leukaemia is more likely to be mentioned on a death certificate than a chronic leukaemia and, in fact, all but one of the acute leukaemias in this study were the underlying cause of death.

It is not known in this study how many of the cancers were registered from death certificates. The average number of years between a cancer registration and death was 1.8 years. This varied by diagnosis, for example with 57% of the acute myeloid leukaemias having the same year of diagnosis and death and 24% having only 1 year difference (overall average 0.8 years). This is in contrast to chronic lymphatic leukaemia, which had 47% with a difference of over 4 years (overall average 3.7 years).

Obtaining cancer registrations in addition to death certificates enables case–control studies to be carried out with incident cases as the event, rather than death. In addition, terminating the duration of exposure at death rather than at diagnosis, particularly if death occurs while the case is still in employment, may lead to an overestimation of the relevant exposure to the hazard under investigation.

Comparison of the diagnoses obtained from death certificates and cancer registrations identified several discrepancies, with a general tendency for the histology to confirm the diagnosis on the death certificate. In many instances, this was the more specific diagnosis. This indicates that, although leukaemia may be registered at an early stage, the registration may not be updated after investigations have established the specific type of leukaemia.This may occur because (a) the new information is not given to the registry or (b) a system may not be in place at the registries to routinely search for changes in previously received diagnoses.

The implications of non-identification of cases and misclassification of diagnosis for this type of occupational study are potentially serious. Previous studies investigating the possible association of leukaemia with exposure to benzene have had only small numbers of cases (Bond et al, 1986; Rinsky et al, 1987; Schnatter et al, 1996). The current study, with relatively large numbers, had the potential to increase the precision of previous risk estimates. It was also important to obtain a correct diagnosis so that leukaemic subtype analyses could be carried out, particularly for acute myeloid leukaemia, the subtype particularly associated in previous studies with exposure to benzene (Aksoy, 1988; McMichael, 1988; Wong, 1995).

There were difficulties in obtaining the histological information, with a disappointingly low number of histology reports received. Identification of the hospitals at which the diagnosis was made was not straightforward, particularly for those who had died at home and for those for whom a cancer registration was not available. Many hospitals did not keep their records as far back as 1951, hence the low response for deaths before 1971. There were also several refusals from hospitals to examine records, the main reasons given being lack of staff and pressure of work.

This study, although small, has shown the importance in nested case–control studies of rare diseases of using both death certificates and cancer registrations for identification of the maximum number of cases. It has also demonstrated the usefulness of obtaining histological records to confirm the diagnosis and to resolve any discrepancies between death certificates and registration. The study identified several areas of incompleteness in the cancer registration system, the most important being the lack of updating of records when new information on the diagnosis had become available. Many regional cancer registries do routinely update their records as they become aware of corrections or additions to their data, and these amendments should also be passed on to OPCS. However, it is not clear how many seemingly minor updates, for example involving the fourth digit of the ICD code, are transmitted. For many disease groups, this may not be important. For diseases, such as leukaemia, for which the fourth digit indicates very different diseases with different aetiologies, treatment and prognosis (Linet, 1985), this level of accuracy is desirable. Future comparative analyses of the diagnoses on the death certificates and cancer registrations for other cancers in the cohort of oil distribution centre workers may provide further insight into other areas of potential discrepancy. The cancer registration system would benefit from the establishment of a registry-wide procedure for ensuring that registrations are updated when new diagnostic information becomes available. Some cancer registries already use histopathology reports as a source of both notification and confirmation of the diagnosis. As the majority of cancers will have some form of histopathology report, all cancer registries should be encouraged to use these.

### ACKNOWLEDGEMENTS

The flagging of the distribution centre cohort was funded by the Institute of Petroleum. We are grateful to OPCS for the provision of the death certificates and cancer registration. We would like to thank Ms Sarah Grace and Mrs Joyce Gilbert for their assistance in

© Cancer Research Campaign 1997

obtaining the histological records and management of the study databases and Dr David Jenkins for examining the histopathology slides.

#### REFERENCES

Aksoy M (1988) *Benzene carcinogenicity*. CRC Press: London

Aksoy M (1989) Hematotoxicity and carcinogenicity of benzene. *Environ Health Perspect* **82**: 193–197

Alderson MR (1988) *Mortality, Morbidity and Health Statistics*. Camelot Press: Southampton

Alexander FE, Ricketts TJ, McKinney PA and Cartwright RA (1989) Cancer registration of leukaemias and lymphomas: results of a comparison with a specialist registry. *Commun Med* **11**: 81–89

Bond GG, McClaren EA, Baldwin CL and Cook RR (1986) An update of mortality among chemical workers exposed to benzene. *Br J Ind Med* **43**: 685–691

Bowie C (1987) The validity of a cancer register in leukaemia epidemiology. *Commun Med* **9**: 152–159

Brandt L, Nilsson PG and Mitelman F (1978) Occupational exposure to petroleum products in men with acute non-lymphocytic leukaemia. *Br Med J* **4**: 553

Kardara M, Acquilla S, Forster D, McCarthy C and Stevenson J (1995) Establishing baseline data in cancer registration in northern England: implications for Health of the Nation targets. *J Epidemiol Commun Health* **49**: 150–152

Kemp IW, Stein GJ and Heasman MA (1980) Myeloid leukaemia in Scotland. *Lancet* **4**: 732–734

Lewis SJ, Bell GM, Cordingley N, Pearlman ED and Rushton L (1997) Retrospective estimation of exposure to benzene in a leukaemia case–control study of petroleum marketing and distribution workers in the United Kingdom. *Occ Env Med* **54**: 167–175

Linet MS (1985) *The Leukaemias – Epidemiologic Aspects*. Oxford University Press: New York

McMichael AJ (1988) Carcinogenicity of benzene, toluene and xylene: epidemiological and experimental evidence. In *Environmental Carcinogens: Selected Methods of Analysis and Exposure Measurements*, Fishbein L and O'Neill IK. (eds), pp. 3–18. IARC: Lyon

OPCS (1987) *Cancer Statistics Registrations 1987*. Government Statistical Service: London

Rinsky RA, Smith AB, Hornung R, Filloon TG, Young RJ, Okun AH and Landrigan PJ (1987) Benzene and leukaemia – an epidemiologic risk assessment. *N Engl J Med* **316**: 1044–1050

Rushton L (1993) Further follow-up of mortality in a UK oil distribution centre cohort. *Br J Ind Med* **50**: 561–596

Rushton L and Alderson MR (1983) An epidemiological survey of oil distribution centres in Great Britain. *Br J Ind Med* **40**: 330–339

Rushton L and Romaniuk HM (1997) A case–control study to investigate the risk of leukaemia associated with exposure to benzene in petroleum marketing and distribution workers in the United Kingdom. *Occ Env Med* **54**: 152–166

Schnatter AR, Armstrong TW, Nicolich MJ, Thompson FS, Katz AM, Heubner WW and Pearlman ED (1996) Lymphohematopoietic malignancies and quantitative estimates of benzene exposure in Canadian petroleum distribution workers. *Occ Env Med* **53**: 773–781

Silcocks PB, Thornton-Jones H and Skeet RG (1989) Making cancer statistics more informative: measures of the quality of recorded diagnosis in a population-based registry. *Eur J Cancer Clin Oncol* **25**: 1467–1473

Swerdlow AJ (1986) Cancer registration in England and Wales: some aspects relevant to interpretation of the data. *J Royal Stat Soc Series A* **149**: 146–160

Vigliani EC (1976) Leukemia associated with benzene exposure. *Ann NY Acad Sci* **271**: 143–151

Wong O (1995) Risk of acute myeloid leukemia and multiple myeloma in workers exposed to benzene. *Occ Env Med* **52**: 380–384

*© Cancer Research Campaign 1997*